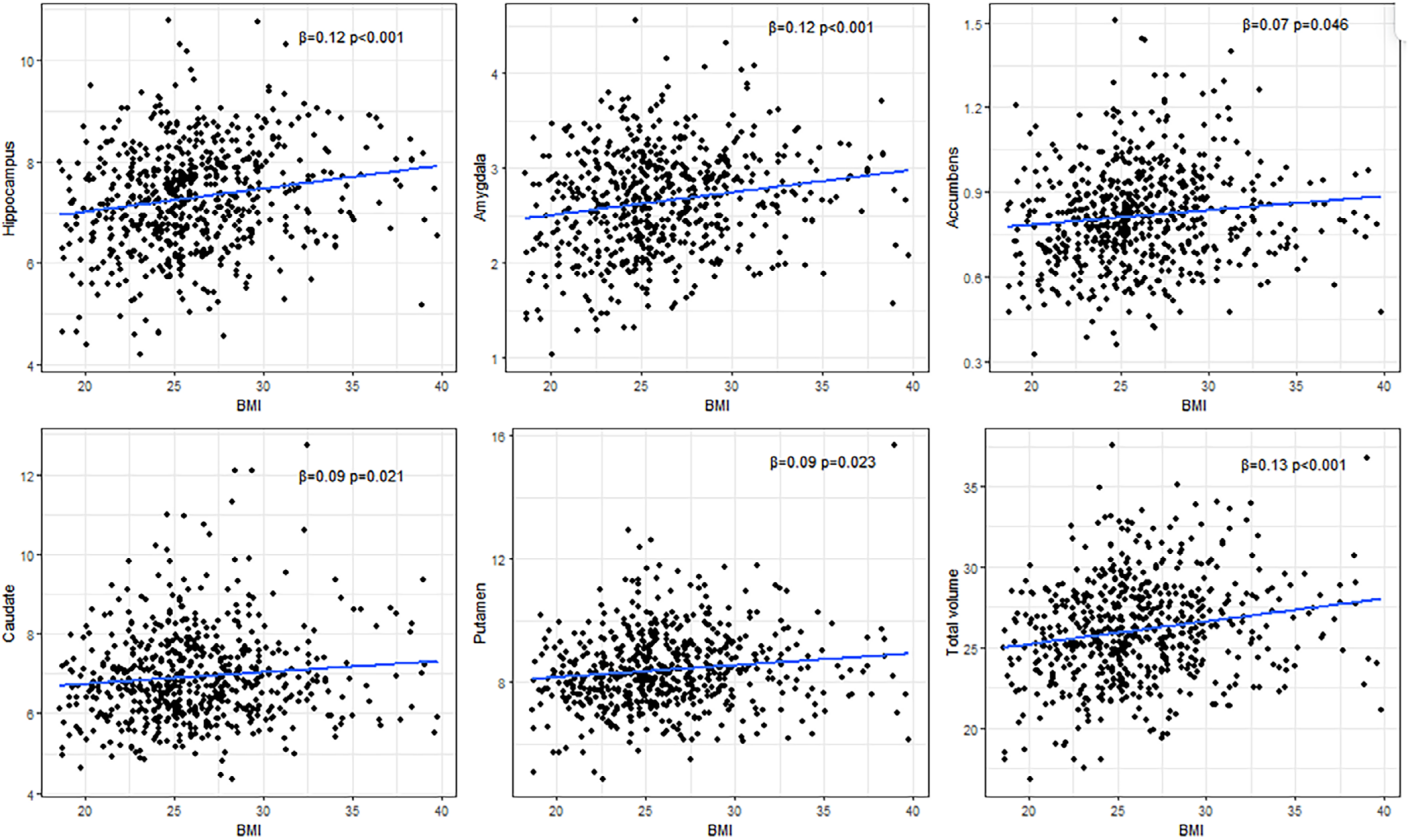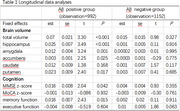# The association of body mass index with the mesolimbic system and cognition in Alzheimer’s disease

**DOI:** 10.1002/alz.084088

**Published:** 2025-01-03

**Authors:** Qin Li, Xiaofeng Li

**Affiliations:** ^1^ The Second Affiliated Hospital of Chongqing Medical University, Chongqing China; ^2^ The Second Affiliated Hospital of Chongqing Medical University, Chongqing, Chong Qing China

## Abstract

**Background:**

The mesolimbic system plays a crucial role in weight regulation and cognition. Previous studies suggest that the pathology of Alzheimer’s disease (AD) can lead to the atrophy of the mesolimbic system and body mass index (BMI) decline. It remains unknown whether BMI is associated with the the mesolimbic system in AD.

**Method:**

The data used in this study was obtained from the Alzheimer’s Disease Neuroimaging Initiative (ADNI) database. All participants underwent at least one Aβ PET imaging. PET imaging was carried out concurrently with brain MRI data and comprehensive neuropsychological assessments. Follow‐up data were collected at 2, 4, and 6 years. Hippocampus, amygdala, accumbens, caudate, and putamen were selected as regions of interest (ROIs) in the mesolimbic system. Linear regression was conducted to assess the relationship between BMI and cognition, and the volume of the ROIs. Linear mixed‐effects models were employed for longitudinal data analysis.

**Result:**

A total of 1182 participants were included in this study, including 608 cases of Aβ positive and 574 cases of Aβ negative. (1) Compared with the Aβ negative group, the Aβ positive group exhibited a decreased volume of hippocampus, amygdala, accumbens, and putamen and lower BMI (P < 0.01); (2) BMI was associated with baseline hippocampal volume (β = 0.12, P < 0.001), amygdala volume (β = 0.12, P < 0.001), accumbens volume (β = 0.07, P = 0.046), caudate volume (β = 0.09, P = 0.021), and putamen volume (β = 0.09, P = 0.023) in Aβ positive group, whereas, in the Aβ negative group, BMI was not associated with the volume of ROIs; (3) BMI was associated with baseline Mini‐Mental State Examination (MMSE) z‐score (β = 0.09, P = 0.002) and memory composite score (β = 0.07, P = 0.015), whereas, in the Aβ negative group, BMI was not associated with cognition; (4) longitudinal data analyses involving Aβ positive participants indicated that BMI was associated with the volume of hippocampus, amygdala, accumbens, putamen, MMSE z‐score, and memory score.

**Conclusion:**

A lower BMI was associated with smaller volume of mesolimbic system and poorer cognition in AD patients.